# Improving access to mental health care in an Orthodox Jewish community: a critical reflection upon the accommodation of otherness

**DOI:** 10.1186/s12913-017-2509-4

**Published:** 2017-08-14

**Authors:** Phil McEvoy, Tracey Williamson, Raphael Kada, Debra Frazer, Chardworth Dhliwayo, Linda Gask

**Affiliations:** 1 Salford, Six Degrees Social Enterprise CIC, Southwood House, Regent Road, Salford, M5 4QH United Kingdom; 2 University of Salford, School of Nursing, Midwifery, Social Work & Social Sciences, Mary Seacole Building, Frederick Road Campus, Salford, M6 6PU United Kingdom

**Keywords:** Underserved populations, Religious minorities, Orthodox Jewish community, Access, Mental health services, Otherness, Dialogic engagement

## Abstract

**Background:**

The English National Health Service (NHS) has significantly extended the supply of evidence based psychological interventions in primary care for people experiencing common mental health problems. Yet despite the extra resources, the accessibility of services for ‘under-served’ ethnic and religious minority groups, is considerably short of the levels of access that may be necessary to offset the health inequalities created by their different exposure to services, resulting in negative health outcomes. This paper offers a critical reflection upon an initiative that sought to improve access to an NHS funded primary care mental health service to one ‘under-served’ population, an Orthodox Jewish community in the North West of England.

**Methods:**

A combination of qualitative and quantitative data were drawn upon including naturally occurring data, observational notes, e-mail correspondence, routinely collected demographic data and clinical outcomes measures, as well as written feedback and recorded discussions with 12 key informants.

**Results:**

Improvements in access to mental health care for some people from the Orthodox Jewish community were achieved through the collaborative efforts of a distributed leadership team. The members of this leadership team were a self-selecting group of stakeholders which had a combination of local knowledge, cultural understanding, power to negotiate on behalf of their respective constituencies and expertise in mental health care. Through a process of dialogic engagement the team was able to work with the community to develop a bespoke service that accommodated its wish to maintain a distinct sense of cultural otherness.

**Conclusions:**

This critical reflection illustrates how dialogic engagement can further the mechanisms of candidacy, concordance and recursivity that are associated with improvements in access to care in under-served sections of the population, whilst simultaneously recognising the limits of constructive dialogue. Dialogue can change the dynamic of community engagement. However, the full alignment of the goals of differing constituencies may not always be possible, due the complex interaction between the multiple positions and understandings of stakeholders that are involved and the need to respect the other’-s’ autonomy.

**Electronic supplementary material:**

The online version of this article (doi:10.1186/s12913-017-2509-4) contains supplementary material, which is available to authorized users.

## Background

### Introduction

The English National Health Service (NHS) has significantly extended the supply of evidence based psychological interventions in primary care for people experiencing common mental health problems [1–3]. Yet despite extra resources, the accessibility of services for ‘under-served groups’ continues to raise concern. The proportion of patients accessing primary care based mental health services from ethnic and religious minority groups, is considerably short of the levels of access that may be necessary to offset the health inequalities created by their different exposure to services, which results in negative health outcomes [4, 5].

Mental health services tend to be used more frequently when they are perceived to be necessary, affordable, acceptable and accessible by the people who use them [6]. When ethnic and religious minority groups are involved as active partners, their levels of engagement, experiences and outcomes generally improve [7]. This is thought to be a result of service providers developing greater cultural competency and actively harnessing the skills and expertise of the people from minority groups who use their services [8]. Improving cultural competency can help mental health workers to establish a more sophisticated understanding of a particular community’s idioms of distress and work with the explanatory models that they use to make sense of mental illness and distress [9, 10]. Drawing upon a community’s resources to co-produce services can also increase community participation and an under-represented community’s sense of control [11]. However, much of the literature on community engagement has a normative tone that is influenced by the logic of instrumental rationality [12]. It tends to portray communities as homogenous entities [13] and tacitly implies that improving community engagement is likely to be beneficial in helping to resolve social issues for which there may not necessarily be an optimal solution that is satisfactory to all of the relevant stakeholders [14]. The literature also tends to understate the complex processes that are involved in developing culturally sensitive services that are able to accommodate the beliefs, values and traditions of ethnic or religious minority groups who seek to maintain a distinct sense of cultural otherness [15–19]. These issues are important to address because efforts to achieve collaborative engagement with minority communities may lead to unfulfilled expectations [20, 21]. They may undermine a community’s sense of internal cohesion by challenging the community’s assumptive world and undermining the sense of fellowship and belonging that closely knit groups of people may share [22, 23].

This paper offers a critical reflection upon an initiative that sought to improve access to NHS funded mental health services within an Orthodox Jewish community. It focusses on two themes of inquiry. Firstly, how the character of the relationships that the mental health service providers established with the community affected the development, implementation and uptake of the initiative. And secondly, how the challenge of finding acceptable ways of accomodating their otherness, through the negotiation of culturally legitimate forms of practice influenced the community’s openess towards the initiative. Otherness is a slippery concept that has been defined in alternative ways by authors who adopt different paradigmatic positions [24–27]. For the purposes of this paper it has been defined in an inter-subjective sense, as being an epistemic perception of seperateness which is located in identified points of difference at an individual (self and other) or collective (us and them) level.

### Jewish identity

Approximately one in 200 people in the United Kingdom (UK) identify themselves as Jewish [28]. Like other ethnic and religious constructs Jewish identity is not a natural category with a clear genetic, religious or socio-cultural basis [29]. It is a dynamically changing and contested construct that has been shaped by religious and ideological schisms, as well as changes in ways of describing ascribed characteristics and patterns of migration, which have led to the formation of cross cultural hybrids [30]. Four broad social groups can be distinguished within the UK Jewish Community: (1) the Haredi community, (2) the Orthodox community, (3) the Liberal, Reform and Conservative communities and (4) non-affiliated and secular people who identify themselves as Jewish (see Table 1). These groups have adopted different approaches towards acculturation [31], which have influenced their levels of exposure and degree of participation in ‘mainstream’ UK culture.

**Table 1:** Affiliations within the British Jewish Community

Haredi	A rapidly growing group that adheres strictly to the corpus of religious law called the *Halacha.* In 2011, proportion of Haredi Jews was estimated to be no more than 10% across the United Kingdom. However, the area in which the project was conducted contains one of the largest concentrations of the Haredi community in the country [100]. The residential preferences of the people of the Haredi community are strongly influenced by their desire to belong to a *kehilla*, a local and autonomous group of people who share their theological outlook, culture, and traditional attitudes towards religious observance [101]. The Haredim follow the guidance of their religious leaders, who act as spiritual guides and educators and their religious ideology regulates the observance of the Sabbath, the preparation of *kosher* foods, social interaction between men and women and many other aspects of their day to day lives. Cultural boundaries are symbolically manifested by a distinctive dress code. Haredi men are often bearded, wear a *kipah* (skull-cap) or a black hat and a formal suit. Haredi women dress modestly, wearing mid to long lengths skirts and they tend to keep their head covered. Men may devote their time to religious studies and stay in religious seminaries called *yeshivas* until their mid to late 20s. Couples tend to marry young and have large families. The Haredim’s level of participation in the labour market outside the internal economy of the community are lower than other Jewish religious groups [102].
Orthodox	Approximately 60% of people in the United Kingdom who identify themselves as Jewish currently consider themselves to be members of the Orthodox community, but the community is steadily declining and the age profile of the community is getting older [100]. The community is more pragmatic in their attitudes towards religious observance than the Haredi community [103]. The people of the community also have greater freedom to make personal decisions, without consulting their religious leaders and the community is more integrated within mainstream British culture [104].
Liberal,Reform and Conservative	A small minority who adopt the position that it is necessary for the Jewish people to reinterpret and adapt their religious culture in order to engage and integrate themselves, within the modern world. For example, the Reform movement departed from the Orthodox tradition, by abandoning the historical prohibition that had prevented the children of Jewish fathers who we born to non-Jewish mothers, being accepted as Jews without a conversion ceremony.
Non-affiliated and Secular	Appromately 30% of Jewish people define their identity in heterodox terms [100, 105]. Many participate in religious activities, but their motivation is due more to their desire to maintain cultural and familial traditions, rather than a strong sense of religious obligation [103]. For some their sense of Jewish identity is not connected to their religious faith and some are overtly anti-religious [106]. They associate via loosely structured, geographically dispersed family and social networks.

### The setting

The City of Salford, Greater Manchester has a population of 234,000 and is among the 10% most deprived Local Authority areas in England [32]. The majority (91.5%) of the population are classed as white British, Irish or other. Within this white population the Jewish community is a significant religious minority. It has increased by nearly 50% over the past decade, from 5200 to 7770 people, due to the rapid expansion of the Haredi community [28].

Within the Haredi community there are relatively high levels of mental health morbidity associated with poverty, unemployment, poor housing and a tendency for some individuals to engage in obsessively stringent religious behaviours [33]. The sense of shame and embarrassment associated within mental health issues can be acute and there is a residual fear that potential marriage prospects will be harmed if it becomes known that a potential marriage partner has had a mental health problem [34–36]. For many people within the Haredi community engagement with a mental health service provider funded through the NHS also entails the crossing of a social boundary [37, 38] that they would not normally transgress without the blessing and permission of their Rabbis [39]. Haredi Rabbis can be ambivalent about mainstream mental health services. On the one hand, they accept that it is necessary in certain circumstances for people to gain access to vital resources and expert knowledge from outside the community in order to meet their needs [40]. Conversely, they are also wary of humanistic ideologies that present radical challenges to their social and cultural order [41]. In our experience a majority of Rabbi’s have strong misgivings about referring people to non-Jewish mental health workers and most would prefer to recommend a worker from the Haredi or Orthodox community who is of the same gender.

### The project

The initiative reflected upon here is the *Eis Ledader* (which means ‘time to talk’ in Hebrew) project. The aim of this project was to address the low take up of NHS funded mental health services within the Jewish community. The project was developed by the local third sector organisation, Six Degrees Social Enterprise with representatives from the Orthodox Jewish and Haredi community organisations, primary care, the NHS and local authority commissioning sectors. The project ran alongside a NHS funded Improving Access to Psychological Therapies (IAPT) service which formed part of a national programme designed to improve access to evidence based psychological therapies in England, by reducing waiting times for people with common mental health problems such as depression or anxiety related disorders. The Salford IAPT service was commissioned by the local Clinical Commissioning Group (CCG) for Salford citizens [42] and delivered by Six Degrees Social Enterprise in its capacity as a service provider.

Prior to the development of the *Eis Ledader* project, the Orthodox Jewish community in the City of Salford had very little history of utilising the IAPT service. During the years 2008 to 2011 an average of less than five Jewish residents in Salford accessed support from the service per year. This was in marked contrast to the number of referrals from other minority groups such as people of South East Asian heritage (average of 72) who make up a very similar proportion of the Salford population. As the *Eis Ledader* project became established, the numbers of Jewish residents accessing the IAPT service increased to 47 people in 2012 and 51 people in 2013. The recovery rate for those people from the Jewish community who completed treatment during this time period was 50%, which was above the national average for IAPT services at that time (44%) [43]. The project has been commended as an example of good practice by the Co-operative Councils Innovation Network and Positive Practice Collaborative for the impact it has had in helping to:

- Break down the stigma of poor mental health within these communities;
- Train a workforce of mental health workers from the community;
- Ensure that the levels of engagement with mental health services within the Orthodox Jewish and Haredi communities are commensurate with other communities in the City of Salford.

## Methods

### Theoretical approach

This critical reflection has been informed by the philosophy of critical realism and the work of the Access to Mental Health in Primary Care (AMP) Study Group [44] which one of the authors belongs to (LG). Critical realism is a philosophical stance that combines a realist ontological position with a relativist epistemological perspective [45]. Critical realists maintain that we live a multi-layered world in which generative mechanisms operate beneath the level of surface (observable) appearances. These mechanisms cannot be observed directly but they can be inferred from the partial regularities that tend to emerge within the context of specific sets of social and institutional arrangements [46]. However, critical realists also acknowledge that our knowledge and understanding of these mechanisms is inevitably partial, as it is constrained by the subjective lens of our discursive narratives, interests and concerns [47, 48].

The AMP Study Group has sought to develop a better understanding of the social processes through which people from minority communities identify the need for and make use of primary care based mental health services. Their research suggests that in order to deliver effective interventions it is necessary to focus on community engagement, the quality of interactions in primary care and the development and delivery of tailored psychosocial interventions [49–54]. Three generative mechanisms have been highlighted by this modelling work; candidacy, concordance and recursivity. *Candidacy* [55] describes how people’s eligibility for healthcare is negotiated in the context of multiple influences which include the social cultural boundaries between different groups, the political economy of resource allocation and the way in which services are institutionally configured. *Concordance* [56] conveys the idea that if health service providers are to move away from ‘doing for’ to ‘working with’ patients it is essential to establish mutually agreed goals and engage in shared decision making. The concept can be applied both at an individual level and at a group level. *Recursivity* [57] refers to the interdependency between the experiences that people have when they receive health services and the subsequent actions they take. These mechanisms help to explain why people from under-represented minority groups perceive the need for mental health services and the contingent events that are likely to determine whether they are likely to make use of those services or not.

### Sampling and data collection

A critical reflective approach [58] was adopted througout the *Eis Ledbader* project. It sought to identify how and why the project developed as it did by identifying mechanisms that were involved, as well as the contingent events and circumstances that influenced the way in which the mechanisms operated.

A combination of qualitative and quantitative data was drawn upon including naturally occurring data, observational notes, e-mail correspondence, routinely collected demographic data and anonymised clinical outcomes measures. Key informants were purposively sampled using stakeholder mapping techniques [59] and included:

- two Rabbis,
- the leaders of two Jewish community organisations,
- a service user,
- the practice manager from a General Practitioner (GP) practice that served the Orthodox Jewish community,
- an independent Jewish mental health practitioner,
- a Public Health consultant,
- the Managing Director of the mental health service provider (Six Degrees),
- a clinical supervisor,
- two Psychological Wellbeing Practitioners.

All of these individuals made up the distributed leadership team that underpinned the project. Written feedback was obtained from four key informants and recorded discussions were undertaken with eight informants and transcribed. The recorded discussions were conducted by two independent researchers either face to face or over the telephone, using a semi structured question schedule developed from the literature (see Additional file 1 for discussion guide). The researchers sought to understand the participant’s point of view by using a combination of open-ended questions, (“Tell me about…?”), as well as prompts (“You said a moment ago…can you tell me more?”).

### Ethics approval

Ethics approval was not sought as no primary research was being undertaken. Following National Research Ethics Service (NRES) guidelines, the project was self-assessed as being a service development. NRES approval is not required when no primary research is being conducted. However as research methods were used, good ethical practices were followed including informed written consent for participation and processes to maintain confidentiality, anonymity and safe storage of data. The final text of the paper was ‘ethically proof read’ [60], by an independent practitioner with research expertise, in order to ensure that any conflicts that arose between maintaining the authenticity of the text, protecting confidentiality and taking care to safeguard the reputation of the community were resolved in an ethically responsible way. The narrative was edited in order to preserve informant anonymity as far as possible.

### Data analysis

The implementation of the *Eis Ledader* project was tracked over a period of 4 years from its initial development A detailed line by line analysis of the discussion transcriptions was carried out and the data was coded [61] using MaxQDA software. The analysis and development of emergent themes proceeded iteratively as the analysts (PM & LG] sought to tease out the processes that were occurring, why they may have been occurring in that context at that time and the impact that they were having at each stage of the project’s life cycle. Six levels of analysis were focused on: (1) the people, organisations and communities that were involved, (2) the language, concepts and logics of justification they used, (3) the perceptions they had about their relationships with their collaborators and non-collaborators, (4) the contextual factors that facilitated or constrained their ability to exert control over the situations they faced (5) their salient concerns and (6) the outcomes of both collaborative and non-collaborative processes as they unfolded over time.

## Results

There were three overarching themes; establishing an arms length relationship, building a collaborative partnership and building a mature collaborative partnership.

**Fig. 1:**
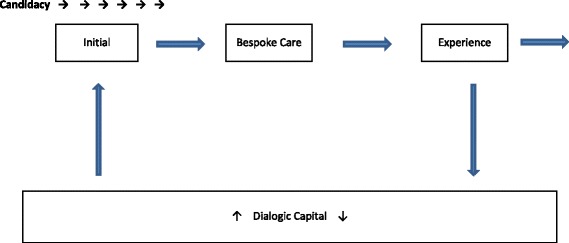
Arms Length Relationship

1. Establishing an Arms Length Relationship (see Fig. 1)

### A disconnection from statutory mental health services

In the Haredi community, in particular, there were virtually no attempts by the people of the community to try to access NHS funded mental health care.

*‘We understood that because they were doubtful of the provision that was already being offered, they preferred to either do things through their GP or go to private providers which is exorbitantly expensive and they were also worried about people within the community finding out about any mental health issues. To my mind, stigma’s a real issue.’* [Public Health Consultant]

There was ‘*a lack of trust’* of existing statutory services within the more orthodox sections of the community. The GPs working closely with this population were also reluctant to refer, and very overworked, referring only the most severely mentally ill people:

*‘They're extremely protective of the patients that they cared for and they were wanting to ensure that nobody was identified or having a label as having a mental health problem that could possibly be avoided.’* [Managing Director]

The gap, for people with common mental health problems, was filled by social organisations within the community providing support but who nevertheless lacked specific mental health expertise. There was recognition that this was an area of social inequality that needed to be addressed with a need to develop more culturally acceptable and sensitive interventions. However there was no clear vision, consensus or strategy in place to tackle the issue.

### Arms length relationships

A loosely distributed network of arms length relationships evolved over an extended period (3 years) between a distributed leadership team who were situated in primary care, Jewish community organisations, social enterprises, and the NHS and local authority commisioning sectors. This leadership team was a self-selecting group of stakeholders who had a combination of local knowledge, cultural understanding, power to negotiate on behalf of their respective constituencies and expertise in mental health care. The team did not meet together on a formal basis but maintained a dialogic form of engagement [62] in the sense that they continuously talked to each other over the project’s lifetime, within the context of a search for some kind of a rapprochement that could facilitate more accessible mental health care for the Jewish community [63, 64]. Although this dialogic engagement, did not result in any immediate agreements or collaborative action, it did help to facilate mutual learning and understanding. For those who were involved this often seemed like a slow, uncomfortable process and it did not seem to generate many tangible benefits. For example, the Six Degrees Social Enterprise as mental health service provider initially tried to work directly with GPs in the Orthodox community but found it difficult to overcome those GPs’ reluctance to refer their patients to the IAPT service. This was evident in the comments of a Practice Manager who voiced frustration about inaccessiblity of NHS funded mental health services, which were free at the point of delivery and the social inequality that restricted access to talking therapies to those within the community who could afford to see a private therapist.

*‘There was the need that wasn't being met, and there was the fact that* [Jewish] *people were having to pay* (privately) *and everybody else got it for free. And that really, really annoyed me. It wasn't that the NHS wasn't prepared to provide it.’*[Practice Manager]

At the same time there was continuing concern about the limitations of the support that *was* available from within the Jewish community:

*‘We had a plethora of organisations who were delivering quasi-mental health support/family support work. And I had very, very strong concerns about the efficacy and the quality of that kind of support, and the fact that some of these organisations were referring to private mental health providers for complex things. And people were completely out of the NHS psychology services loop. I thought that was quite* dangerous.’ [Community Leader]

### Developing relationships

Personal relationships were established and maintained between the employees of the mental health service provider and the other stakeholders both from within and outside the Jewish community through informal conversations. This generative dialogue [65] helped to create a climate of greater trust and confidence. As stakeholders from within the community sensed that the service provider understood why they approached the subject of candidacy in the way that they did, they felt less vulnerable. They became more receptive to new ideas and they engaged in preliminary discussions and how their cultural resources could be mobilised and used, in a way that allowed the people of the community to retain the sense of control that they valued.

*‘I wasn't that precious about whether we owned it or not. I wanted to build up the capacity of the people in the community to support people of their own community.’* [Managing Director]

The organic development [66, 67] of these arms length relationships promoted the pooling of ideas about ways of addressing issues of mutual concern and stimulated exploratory discussions about how the various actors could potentially work together. A key milestone for the development of the notion of appropriate candidacy for ‘mental health care’ within the community was the establishment of the STEPS® course [68]. This was a bespoke care pathway incorporated within the IAPT service, as a means of addressing mental health issues in ways that were not seen a stigmatising but as ‘self-improvement’, building upon community values and providing the option for self referral. This illusrates the central role of language in describing the content of the care pathway in a way that had cultural legitimacy:

*‘Self improvement is a really common sort of idea and it’s the theme which people would encourage others to do, so in synagogues and in Jewish lectures the idea would come in on a regular basis. So to advertise a course using a self improvement message or to talk about coming to therapy for self improvement is absolutely fine. Wellbeing is also okay but perhaps less so; and then anxiety and depression you really want to stay away from those sort of words.’* [Independent Mental Health Practitioner]

The course was sponsored by Six Degrees Social Enterprise but was run by a community-based organisation and faciltated by an independent Orthodox Jewish mental health worker. Due to his local knowledge, the worker understood the subtle differences in attitudes towards the mental health services between the different sub-communities and was senstive to their specific needs.

*‘X* (the Jewish mental health worker) *has been fairly integral to the life of the project but not in a managerial way, just as a person that's from the community, trusted by the community and has quite a high level of expertise as a therapist ’* [Managing Director]

Following the suggestion of the coomunity leaders the course initially aimed to attract young males, especially those on the edge of the community who might not be ‘fitting in’ and potentially had low self-esteem. However, it actually recruited a much broad demographic of males from across the various sub-sectors of the community.

**Fig. 2:**
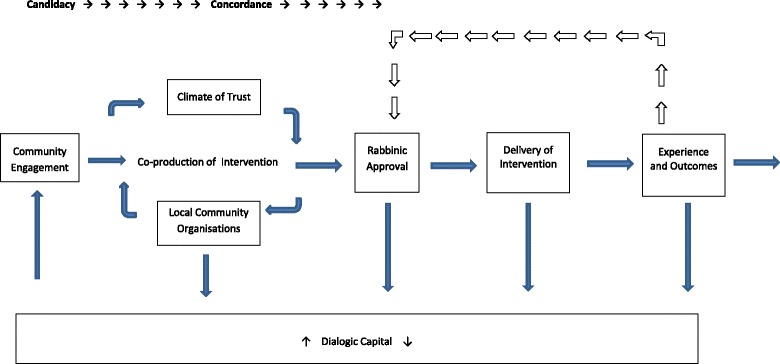
Collaborative Partnership

2. Building a Collaborative Partnership (see Fig. 2)

### Engaging the community

The project sought to develop a service that was more *concordant* with the community’s values by employing two trainee Psychological Wellbeing Practitioners from the community whose posts were jointly funded by the local CCG and Health Education England.

*‘Recruiting the workers in the community and developing capacity from the community I think has been a key part of the success of it.’*[Community Leader]

The Psychological Wellbeing Practitioners from the community specifically set out to establish closer links with local Rabbis and community agencies.

*‘The Rabbis have been really kind of important and necessary to get it going’.* [Psychological Wellbeing Practitioner]

‘*Previously people were not confident that the professionals they would be referred to would understand the issues and the lifestyle of the Orthodox Jewish community. For them to have confidence, it was necessary for professionals to have a thorough knowledge and understanding of these matters and an appreciation and sensitivity to them. We were being approached by people who have really required this service and were unable to obtain the necessary help….’* [Rabbi]

The approval of the Rabbi was crucially important, as people with problems not only sought the Rabbi’s help but also his advice on where they could obtain specialised assistance.

*‘The way I see it the Rabbi is the one who, if you’ve got a smashing project or a really weak project that’s relevant, the Rabbi is going to be the one who says, yeah, go ahead and use that service, or even if it’s great service I wouldn’t use it if I were you because of X, Y and Z.’* [Psychological Wellbeing Practitioner]

### Engaging clients

To engage people from the community it was essential to provide access to both male and female therapists and to have mental health workers who were familiar with the requirements of Jewish law in relation to social practices such as not touching and the timing of prayers which might occur during therapy. It was also important to be aware of other issues relating the community’s values that stress the importance of marriage and family life:

*‘One of the ladies said it’s a relief not to have to explain my life,…Say you go to your GP who isn’t Jewish, you have a seven minute appointment, you spend five minutes explaining the rules of your religion before you can actually discuss the problem.’* [Public Health Consultant]

There was a particular awareness of the needs of those in the community who might feel isolated as others because they did not ‘fit with the norm’, such as women who were unable to concieve. A decision was made to initiate self-referral to the mental health service in order to try and make the service easier to access for those who may be wary of accessing the service through the established channels.

It was also apparent that the ways in which the interventions were spoken about were an important factor within the contested arena over what forms of *culturally concordant* care and could legitimately be used to try to support the wellbeing of community members that experienced mental health problems. Language was not only an issue in how the service was described, but in the clinical encounter:

*‘There are some key words which are difficult to explain in English, there’s just one word in Hebrew, one or two words in Hebrew which are straightaway understood by a Jewish worker’* [Independent Mental Health Practitioner]

How to ensure maintainence of confidentiality within the community was something which sometimes needed to be discussed with individual’s who were worried about being identified as having mental health problems:

*‘I might say to them, if I see you outside I will ignore you.’* [Psychological Wellbeing Practitioner]

But provision of choice was important. Both Psychological Wellbeing Practitioners were from an Orthodox background, but one was more embedded within this community than the other. Some people preferred *not* to see a person from within the community:

*‘Choice is important, yeah, and we have to also respect that some people from the Orthodox Jewish community might choose not to see someone from their own community, like me personally, would prefer not to see somebody from my own culture, I do like the diversity.’* [Psychological Wellbeing Practitioner]

**Fig. 3:**
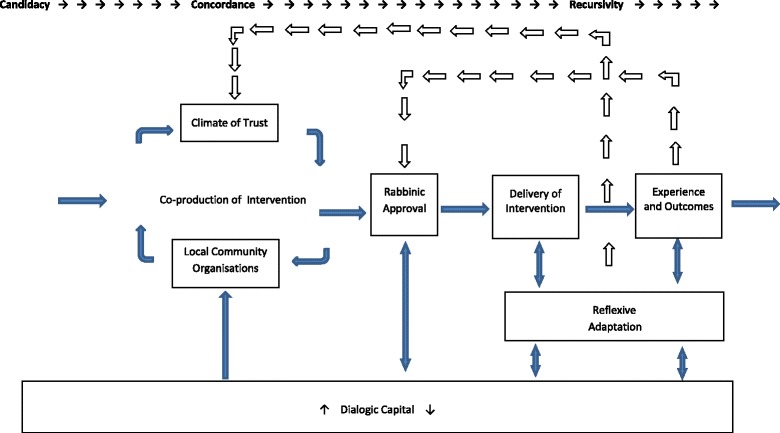
Mature Collaborative Partnership

3. Building a Mature Collaborative Partnership (see Fig. 3)

### Recursive relationships

As the service developed there was a discernible change in perceptions within the community, as the community members became more confident that they would be able to work with the service provider to achieve their goals. The confidence was undepinned by complex and *recursive* relationships between the key stakeholders in the community and the Six Degrees team. This built upon the previous groundwork that had been done to establish dialogue, understanding and working relationships. Some contradictions and tensions remained but these ‘feedback loops’ created more possibilities for action, both in changing the scope of the project and modifying the interventions that were being delivered. Feedback from both the Rabbi and people who used the service was crucial in maintaining trust in the service, but also increasing referral, and there was understandable concern about this:

*‘That person won’t necessarily go to their next-door neighbour and say that I went to a mental health clinic and they’re great, but they probably will tell their Rabbi how it went and the Rabbi will take that on board and say, okay, look, there’s been some positive outcome, let’s see how it goes, and continue referring. And that wheel, if you like, that wheel of word of mouth feedback is really crucial.’* [Practice Manager]

The Psychological Wellbeing Practitioners from the community were exposed to the additional stresses of being embedded within the community, discovering things about their community that they may not have known and meeting people who break taboos within it:

*‘If you employ somebody from a community they are likely to know closely…or not that closely the person and all their friends and family. So it creates tensions around confidentiality and people feel that they're exposed. And also for the workers, seeing their own community with different eyes, because they'll exposed to inherent tensions and problems within their own community which they may not have been conscious of.’* [Practice Manager]

These issues surfaced particularly in clinical supervision (support sessions for practitioners with their manager) when there were occasions when workers debriefed about the difficulty they faced in dealing with the emotional challenges of working with patients and in questioning their own assumptions:

*‘Transference and countertransference that people have to work with, working in that environment is the phenomenally complex and pertinent to what they're doing, where they're dealing with major social sensitivities on both sides…. The sessions would focus on things that people should be adhering to culturally, or as part of their group.. rather than what problems were brought, so the therapy has always been diverted and the practitioner struggled to stay with the problems and goals.’* [Clinical Supervisor]

The challenge of dealing with the stress of never being able to ‘switch off’ due to their own place within that community was highlighted by one of the Psychological Wellbeing Practitioners:

*‘If a non-Jewish worker sees a non-Jewish patient they can just go home, switch off and never see the patient again. For a Jewish worker, and I feel this myself, there’s a sense that actually we don’t quite switch off because they’ll see the patient often…I see a patient often in a synagogue, in a Jewish kosher shop, in the community, so that’s for me been a real, I don’t know, it’s a real…for me it can be quite anxiety provoking. But I suppose I’ve got to envision, I’ve got space to reflect upon it, I’ve spoken about it before and people in my team are aware of it.’* [Psychological Wellbeing Practitioner]

### Evaluating outcomes

The CCG in providing funding for the development project allowed a realistic timeframe for the processes described above to be achieved.

*‘When it came to reporting back to the CCG we’d built planning into the timeframe so it wasn’t a case of here’s the money, set it up, whizz, bang, off you go. We actually built in that time…. to do the development work with the community and working with the Rabbis to get approval.’* [Public Health Consultant]

Routine quantiative clinical outcome measures were collected as for the Improving Access to Psychological Therapies programme (see https://www.england.nhs.uk/mental-health/adults/iapt/). Collecting this data itself posed some problems given the language used in the forms such as the Personal Health Questionniare version 9 (PHQ9) [69]:

*‘because it measures depression and anxiety symptoms, which has not gone down very well when, certainly in supervision, the guys who are embedded within the community, have raised this as a bit of an issue, and we’ve looked at ways around that, and what it’s about. We can’t change the form, because we won’t be able to capture the details, but what we’ve looked at is how we present the information and why we need this information* [Clinical Supervisor]

There was however a degree of realism about what could be achieved in short-term interventions:

*‘I think that we are not quite sure what the outcomes do, but people have had a go-to place where they've been listened to and given the space outside of the pressure pot where they can think things through. …we certainly wouldn't have taken away all the pressures. The most that could've happened is maybe that people as a result of just having what input they've had is find a way of living with it. Because some of the pressures of what I know about are pretty major really.’* [Managing Director]

For some, there was a sense that the process had not developed in the direction they had originally wanted. One of the Rabbis felt strongly that, although the process had been successful in what it had achieved, there remained for him, an enduring sense that the needs of the community were still not being fully addressed. He would have preferred a more comprehensive service which met the needs of the people from the community who had more severe mental health problems:

*‘They might be a very good project, I’m not saying...it probably is a very good project for what they are doing, but it’s not what I was looking for.’* [Rabbi]

One community leader indicated that she felt that more emphasis should have been placed upon the delivery of psychoeducational training to groups within the community:

*‘It hasn't continued in the way I had originally envisaged… I had this vision, which hasn't particularly materialised, in the sense that I wanted that somebody should be going in to each of these different organisations..’* [Community Leader]

And there was disappointment that the family doctors who were most embedded in the community (unlike those on the periphery with a more mixed population) were still reluctant to make referrals to the service. However, there was a general sense that something important had been jointly achieved, and awareness of the process by which this had been done:

*‘You need to have capacity built in, you need to be engaged in leadership, you need to get it right and get acceptability by it. And that's what they did.’*[Community Leader]

### Sustainability

At the time of writing, the process has now reached the point where the *Eis Ledader* project has been embedded and incorporated into the infrastructure of the mainstream service provision, with allocated resources that will enable it to be continued. However, there remains a need to review outcomes achieved, revise targets and consider how the process of engaging with the community can be sustained. There were a range of views about what should be done next, from a further re-engagement with the low referring family doctors, providing them with more feedback, training voluntary workers in local community organisations (as above) to re-launching the STEPS® [68] course as a ‘life-coaching’ project in order to increase take up again.

*‘The service could be almost a defined element of it as life coaching. And I don't think that, as a community, we've yet managed to push that benefit, which other* [Jewish] *communities and other parts of the world have, this life coaching benefit for people.’* [Community Leader]

Finally, there was also recognition of the need to consider how mainstream interventions, offered to the broader community could be culturally adapted too:

*‘When I see mindfulness groups accessible for the wider community, I want to know why we can’t take those into the Orthodox Jewish community as well. Let’s tweak them and make them accessible’* [Practice Manager]

## Discussion

Improvements in access to mental health care for the people of the Orthodox Jewish community were obtained through the collaborative efforts of a distributed leadership team. The team were able to work with the community to develop a bespoke and accessible service that accomodated their otherness. The members of this leadership team had the social capital across the political, technical and experiential domains [70], which may be required to develop effective and inclusive approaches to dealing with complex social issues. They had a combination of local knowledge, cultural understanding, power to negotiate on behalf of their respective constituencies and expertise in mental health care.

Over the project lifetime, the relational work [71] that was cultivated by this distributed leadership team helped to create deliberative capacity [72] that was needed to maintain a dialogue about the issues in hand, without always understanding or sharing each other’s perspectives. The term dialogic is used in a variety of ways but is generally accepted that the goal of dialogic engagement is to facilitate understanding, through the recognition and acceptance of difference and the creation of opportunities for voices to be heard that may have been silenced [73]. Dialogical approaches to engagement differ from conventional approaches that focus upon problem solving, in the sense that they do not necessarily attempt to directly reconcile differences in order to establish a shared position and common ground. The dialogue that was sustained in this project did not produce any immediate dividends, but it demonstrated to the community that genuine efforts were being made to try and understand and accommodate their needs. This helped to establish the level of confidence and control over the project that team members were seeking. Over time, these connections and relationships eventually enabled the team to act opportunistically in mobilising their resources and exploring possibilities for collective action that supported the candidacy of people who sought mental health care.

The recruitment of workers from the community who were able to understand and manage the politics of cultural identity was a crucial confidence building measure that helped the mental health service provider to engage with the community. It clearly signalled to the community that the service provider was respectful of the communities otherness in ways that were concordant with its cultural identity, strengths and its traditions. When set within the context of the work that was done to engage with the community it helped to demonstrate that the service provider understood many of the things that the people of the community most valued. For example, their strong sense of identity, the ethos of mutual help and the feeling of belonging that often served as a buffer in helping to maintain good mental health [74–76]. Yet, despite the progress that had been made, comments such as those from the Rabbi who spoke about his reservations about the project, hinted at the lingering sense of frustration from some within the community that their needs, were still not being fully accomodated.

Negotiating the accomodation of otherness was a recursive process that was carried out in a dynamically shifting and contested terrain, not least because the Jewish community itself is rapidly evolving and has a dynamic of its own. Enabling the community to maintain its separate existence and protecting its cultural mores, whilst simultaneously opening up access to mental health resources that the community could benefit from was a particular challenge for all parties concerned. For the Rabbis within the Haredi community who are entrusted with the role of spiritual leader and interpretor of *Hashem’s* (God’s) laws as laid out in the religious texts of the Torah*,* protecting the communities identity and position within the social order, it was a particular challenge. From their perspective, the project opened up access to the new resources that could have both constructive and destructive potential. In addition to impact that it could have upon the social boundaries that protected the wider community, it also had potential ramifications for the way in which the local sub-communities positioned themselves in relation to each other.

For the service providers and other community stakeholders who initiated and sustained the dialogue, it was a sometimes frustrating and disconcerting process as they sought to develop an understanding of the influence of inter-communal politics, within a community that is neither restricted to the local geographical vicinity or internally homogenous. Cultural sensitivities that produced complex interactions could not have been predicted by a linear project plan.

For the mental health workers that were recruited from the community, the accomodation of otherness exposed tensions, which were sometimes difficult to negotiate, not only because it threatened the distinction between their public and private lives [77], but also because they encountered otherness and separateness, both within themselves and in their own communities. Their experiences have many parallels with those of psychotherapists such as Spero who come from an Orthodox Jewish background. In a series of papers, published in the 1980s and early 1990s, Spero clearly delineated how the religious beliefs and identity of the mental health worker may permeate the transference and countertransference relationship between mental health worker and client [78–81]. This can generate powerful feelings that are linked to the processes of overidentification or rejection, which may stir up internal conflcit that is derived from the unconscious sense of otherness that can resonate within ourselves [82, 83]. Furthermore, he warns that for the mental health worker having inside knowledge, creates the risk that they may presume a shared cultural background that is deeper than that which actually exists [84].

For the people of the Orthodox Jewish community, having the opportunity to seen by a mental health worker recruited from within the community was both an enabler and a constraint. This finding is consistent with the with the work of Kushner and Sher [85, 86] that suggests that the decision to engage with mental health services is influenced by dialectical tension between *avoidance* tendencies such as fears of stigma, negative judgements and concerns about what treatments may involve and *approach* tendencies such as confidence in the ability of professionals to help alleviate distress. The culturally specific understanding and position of the mental health worker within relatively closed communities, may help to diffuse or exacerbate feelings of threat, vulnerability and shame. Different factors are likely to be salient for each individual person, depending upon the particular issues they have, the trajectory of their exposure towards mental health services and their previous life experiences [87, 88].

This reflective paper did not incorporate the voices of those within the Orthodox Jewish community who have not had contact with the *Eis Ledadar* project and the focus upon dialogic engagement that emerged from the critical analysis may have obscured the role that ‘invisible infrastructures’ played as generative mechanisms that enabled dialogue to happen. A more detailed examination of what actually happens during the process of dialogue would be required to obtain a clearer understanding of tensions that may have been suppressed, a more nuanced view of the individual concerns and the impact that context dependent meanings may have upon the trajectory dialogic engagement may take [89]. Subtle differences are often a source of rivalry and potential hostility and the language used to describe the social practices of individuals or groups can have contradictory effects when engaging with different constituencies.

We wish to highlight two issues that illustrate this potential. The first is concerned with the way in which we used the term ‘obsessive’ in a nosological sense [90], to describe intrusive and highly distressing thoughts that can only alleviated by the scrupulous performance of religious rituals. Our use of the term was thought to be acceptable by the members of the Jewish community who we consulted with, but we are also aware that depending upon the context and the sensitivities of those involved, it could be viewed in some quarters as being disrespectful. It is known that non-Orthodox people may be more wary of identifying religious scrupulosity as problematic, than their Orthodox counterparts who are acquainted with the cultural norms within their religious communities, as they are concerned about causing offence [91]. Secondly, we are aware that it could be inferred that we are suggesting that the Haredi community itself was responsible for the low level of access to mental health care, as it chose to maintain the sense of otherness that is an integral part of its cultural identity. This is not the position we take. Our view is that it is necessary to understand the communities culture and social situation and to cultivate dialogue about the barriers to access that are rooted in both real and perceived differences in the framing of the complex issues that are involved in accommodating otherness.

Despite these limitations, this reflective account has highlighted the crucial role that dialogic engagement played in helping the project team understand and relate to a community with a unique religious and cultural background. The ability of the project team to build relationships and engage in constructive dialogue helped the team to move beyond rhetorical commitments [92] and develop mature collaborative partnerships, with the resilience that was needed to handle differences in cultural outlook and points of view. These are aspects and forms of leadership that are often devalued.

## Conclusion

Critical reflections can help us to question and re-examine our assumptions and look at things afresh in order to gain new insights and open ourselves up to new possibilities [93]. This reflection has highlighted the role played by a distributed leadership team, which adopted a dialogic approach to improve access to mental health care provision within an Orthodox Jewish community through the ‘accomodation of otherness’. Within economies of care that are driven by the logic of the market and instrumental rationality, predefined outcomes and targets play a prominent role and it is often presumed that collaboration can be mandated through regulatory regimes or brokered via consensual aggreement between divergent interest groups [94, 95]. Dialogic engagement is an alternative approach based on the cultivation of relational processes that can open up new possibilities for action. It is based upon the view that mature relationships are underpinned by a nuanced appreciation and acceptance of difference, together with an understanding of the dynamics that contribute to a percieved sense of otherness [96–98]. The paper illustrates that dialogic engagement can further the mechanisms of candidacy, concordance and recursivity that are associated with improvements in access to healthcare in under-served sections of the population**,** whilst simultaneously recognising the limits of constructive dialogue. Dialogue can change the dynamic of community engagement. However, the full alignment of the goals of differing constituencies may not always be possible, due the complex interaction between the multiple positions and understandings of stakeholders that are involved and the need to respect the other’s autonomy [99].

## Additional file

Additional file 1: Discussion guide containing details of evaluation brief and interview focus. (DOCX 14 kb)

## Abbreviations

AMP: Access to Mental Health in Primary Care
CCG: Clinical Commissioning Group
GP: General Practitioner
IAPT: Improving Access to Psychological Therapies
MaxQDA: Qualitative data analysis software for mac OS X and windows
NHS: National Health Service
PHQ9: Personal health questionniare version 9
STEPS®: STEPS to Excellence - The Pacific Institute
UK: United Kingdom
