# Supplementary material for: Improving access to mental health care in an Orthodox Jewish community: a critical reflection upon the accommodation of otherness
Source: BMC Health Serv Res. 2017 Aug 14;17:557. doi: 10.1186/s12913-017-2509-4 (PMC5557521; doi:10.1186/s12913-017-2509-4)
Supplement: Additional file 1: — Discussion guide containing details of evaluation brief and interview focus. (DOCX 14 kb) [file 12913_2017_2509_MOESM1_ESM.docx]

**Discussion Guide**

**Evaluation Brief**

1. Explore the processes via which this mental health project was implemented.
2. Explore the subsequent causal mechanisms that have accounted for its outcomes.

**Interview Focus**

1. The inter-organisational processes via which this mental health project was implemented.
2. The influence that the negotiation of cultural legitimacy has had upon the processes through which the Haredi community make use of the project.
3. The relationships that were established between the key stakeholders and how they implementation and uptake of the project.
4. The impact of the investment in and enhancement of the service provision to this community.

**Technique**

Semi structured interviewing is to be used to collect qualitative data by setting up a situation 9the interview) that allows the interviewee the time and scope to think and talk about their opinions on these topics.
